# Whole Exome Sequencing in 26 Saudi Patients Expands the Mutational and Clinical Spectrum of Diabetic Nephropathy

**DOI:** 10.3390/medicina61061017

**Published:** 2025-05-29

**Authors:** Imadeldin Elfaki, Rashid Mir, Sanaa Almowallad, Rehab F. Almassabi, Wed Albalawi, Aziz Dhaher Albalawi, Ajaz A. Bhat, Jameel Barnawi, Faris J. Tayeb, Mohammed M. Jalal, Malik A. Altayar, Faisal H. Altemani

**Affiliations:** 1 Department of Biochemistry, Faculty of Science, University of Tabuk, Tabuk 71491, Saudi Arabia; salmowaled@ut.edu.sa (S.A.); rf-saif@ut.edu.sa (R.F.A.); 2 Prince Fahd Bin Sultan Research Chair for Biomedical Research, Department of MLT, Faculty of Applied Medical Sciences, University of Tabuk, Tabuk 71491, Saudi Arabia; rashid@ut.edu.sa (R.M.); wed3x7@gmail.com (W.A.); azizda@moh.gov.sa (A.D.A.); jbarnawi@ut.edu.sa (J.B.); f.tayeb@ut.edu.sa (F.J.T.); mjalal@ut.edu.sa (M.M.J.); maltayar@ut.edu.sa (M.A.A.);; 3 Metabolic and Mendelian Disorders Clinical Research Program, Precision OMICs Research & Translational Science, Sidra Medicine, Doha 26999, Qatar; ajaz.bhatt@gmail.com

**Keywords:** diabetes mellitus (DM), type 2 diabetes mellitus (T2DM), diabetic nephropathy (DN), diabetes complications, whole exome sequencing (WES), bioinformatics

## Abstract

*Background and Objectives*: Type 2 diabetes mellitus (T2DM) is a health problem all over the world due to its serious complications such as diabetic nephropathy, diabetic neuropathy, diabetic retinopathy, cardiovascular diseases, and limb amputation. The risk factors for T2DM are environmental, lifestyle, and genetic. The genome-wide association studies (GWASs) have revealed the linkage of certain loci with diabetes mellitus (DM) and its complications. The objective of this study was to examine the association of genetic loci with diabetic nephropathy (DN) in the Saudi population. *Materials and Methods*: Whole exome sequencing (WES) and bioinformatics analysis, such as Genome Analysis Toolkit, Samtools, SnpEff, Polymorphism Phenotyping v2, and Sorting Intolerant from Tolerant (SIFT), were used to examine the association of gene variations with DN in 26 Saudi patients (18 males and 8 females). *Results:* The present study showed that there are loci that are probably linked to DM and DN. The genes showed variations that include COCH, PRPF31, PIEZO2, RABL5, CCT5, PLIN3, PDE4A, SH3BP2, GPR108, GPR108, MUC6, CACNA1D, and MAFA. The physiological processes that are potentially affected by these gene variations include insulin signaling and secretion, the inflammatory pathway, and mitochondrial function. *Conclusion:* The variations in these genes and the dysregulation of these processes may be linked to the development of DM and DN. These findings require further verification in future studies with larger sample sizes and protein functional studies. The results of this study will assist in identifying the genes involved in DM and DN (for example, through genetic counseling) and help in prevention and treatment of individuals or populations at risk of this disease and its complications.

## 1. Introduction

Diabetes mellitus (DM) is a metabolic disease in which there is persistent increased blood sugar. The WHO reported that the number of people living with diabetes increased from two hundred million in the year 1990 to eight hundred million in year 2022 [1]. It is estimated that 564 million people worldwide already have diabetes, and that number will rise to 600 million by 2035 and 783 million by 2045 [2]. The prevalence rate of DM in the KSA has been reported to be between 9 and 22% between 1980 and 2008, and there are about 7 million diabetic and 3 million prediabetic Saudi individuals [3].

In general, the types of DM include type 1 diabetes mellitus (T1DM) which is developed due to the immune destruction of the insulin producing pancreatic beta cells by immune cells, the CD4 cells orchestrating the immune attack, and CD8 cells targeting the pancreatic beta cells, leading to hyperglycemia [4]. The second type of DM is type 2 diabetes mellitus (T2DM) resulting from insulin resistance or impaired insulin action in peripheral tissues (liver, skeletal muscles and adipose tissues) and pancreatic beta cell dysfunction [5]. The third type of DM is gestational DM; it is a type of insulin resistance that occurs during the late second trimester or early third trimester of gestation [6].

DM has very serious complications that can be classified as microvascular and macrovascular complications [7]. These complications lead to long-term negative effects on crucial tissues and lead to increased mortality rate [7]. The microvascular complications include diabetic kidney disease or diabetic nephropathy (DN), diabetic neuropathy, and diabetic retinopathy. While the macrovascular complications include cardiovascular, cerebrovascular (stroke), and peripheral artery diseases [7,8].

DN is a serious renal-related complication of T1DM and T2DM, and it is the second important cause of end-stage kidney disease (ESKD) [9,10]. About 40% of DM patients develop DN that affects 700 million people globally [2]. The prevalence of diabetes-related ESKD cases worldwide rose from 19.0% in 2000 to 29.7% in 2015 [2]. In 2021, there were 571.29 million deaths because of DN [11]. The DN can result in elevated blood pressure, edema, and proteinuria [9,10].

In DN, hyperglycemia activates the pathways, oxidative stress, diacylglycerol-protein kinase C, advanced glycation end-products, hexosamine, TNF-α signaling, T-lymphocytes, and metabolic pathways [12,13]. These activated pathways interact with each other, inducing inflammatory reactions in diabetes conditions. The main pathological features of DN are the glomerular basement membrane thickening, glomerulosclerosis, renal endothelial dysfunction, accumulation of mesangial excessive extracellular matrix (ECM), abnormality of podocytes, and tubulointerstitial renal fibrosis [13,14]; nevertheless, the pathophysiology of DN remains to be uncovered [14]. The downstream consequences of DN include premature death due to end-stage renal disease (ESRD) and are associated with increased cardiovascular mortality [15], secondary hyperparathyroidism [16], and anemia [17].

T2DM is developed by the interactions of environmental, lifestyle, and genetic risk factors. The environmental and lifestyle risk factors include obesity, physical inactivity, smoking, stress, and unhealthy diet [18]. Maintaining a healthy body weight, regular physical exercise, smoking cessation, no alcohol, a healthy diet, and blood pressure control prevent or delay the development of T2DM and its complications [18,19,20].

The genome-wide association and exome and genome-wide sequencing studies [21,22,23,24] have revealed the association of certain loci with diseases including DM, DM complications, cardiovascular disease, and cancers [25,26,27]. Nevertheless, understanding the genetics of diabetes and diabetes complications is still required. The objective of the present study was to investigate the association of gene variations with the development of diabetic nephropathy (DN) in Saudi population. The whole exome sequencing (WES) and bioinformatics tools were employed for this purpose. The identification of DN risk loci will assist in prevention, diagnosis, and treatment.

## 2. Materials and Methods

### 2.1. Study Design

This project was ethically approved by the department of research and studies, directorate of health affairs, Taif, approval No. 229, and by the committee of research ethics of the hospitals of armed Forces, Northwestern Region, approval No. R & RE C2016-115. The population comprised T2DM patients visiting the hospitals for routine checkup and dialysis (Table 1). This study included patients with clinically confirmed cases of T2DM and diabetic nephropathy. This study included only citizens of Saudi Arabia, both males and females. All subjects gave informed consent. A standard questionnaire was used to document the socio-demographical characteristics such as age, sex, and lifestyle. This research was conducted to determine genetic variants that are linked to T2DM and its development into diabetic nephropathy. A total of 26 T2DM participants (18 males and 8 females), with DN, were enrolled in this study. All participants gave informed consent. Inclusion Criteria: Adults with T2DM, diagnosed with DN (elevated serum creatinine, increased eGFR, or albuminuria). Saudi patients with other diseases or patients with other complications of DM were excluded from this study.

### 2.2. Sample Collection and DNA Extraction

Peripheral blood (5–10 mL) was drawn from each participant via venipuncture into EDTA-coated tubes. Immediately following collection, the blood samples were stored at 4 °C and processed within 24 h. Genomic DNA was extracted using a standard phenol-chloroform method or a commercially available genomic DNA extraction kit (e.g., Qiagen, Hilden, Germany), following the manufacturer’s protocol. The quantity of extracted DNA was measured by spectrophotometry (e.g., NanoDrop spectrophotometer, Thermo scientific, Massachusetts, USA), and purity was assessed by the A260/A280 ratio. Additionally, DNA was run on a 1% agarose gel to verify integrity before downstream analysis.

### 2.3. Whole Exome Sequencing (WES) and Library Preparation

DNAs with acceptable quality (OD_260/280 ≈ 1.8) from all participants were subsequently subjected to whole exome sequencing (WES). Sequencing was carried out according to a standardized laboratory procedure to guarantee accuracy and reproducibility. Library construction was first performed where the DNAs were indexed and made ready using a quality-tested exome capture platform, e.g., SureSelect Human All Exon V6, Agilent Technologies, California, USA) or an equal platform. The second step was exome capture, which preferentially enriched the coding regions (exons) and their flanking sequences. This step efficiently reduced off-target reads while maintaining high coverage of disease-related loci, thus optimizing data quality for downstream analysis.

After exome capture, sequencing was carried out on an Illumina NovaSeq 6000 platform (San Diego, CA, USA) with 2 × 250 bp paired-end reads. All exome-enriched libraries were combined, and every sample was sequenced to an average on-target depth of ~300×, ensuring adequate read depth for consistent variant calling. To ensure data integrity, strict quality control steps were implemented. Every library was measured with a Qubit fluorometer and tested on an Agilent Bioanalyzer to verify proper fragment size distribution and enrichment efficacy. All these steps cumulatively ensured high-quality sequencing data for future genomic analysis.

### 2.4. Quality Control and Read Alignment

After sequencing, FastQC (version 0.11.9) was employed to determine raw read quality, to detect over-represented sequences, and to recognize other types of artifacts. Trimmomatic (version 0.39 or similar) was utilized with default parameters (e.g., SLIDINGWINDOW:4:20, MINLEN:50) to trim low-quality bases and adapter sequences from the reads. Reads failing to pass a stringent quality threshold (Q-score > 30) were excluded.

High-quality reads from every sample were mapped to the human reference genome (GRCh38.p14) with Bowtie2 (Version 2.5.4) or BWA-MEM2. The default settings were used to allow for rapid gapped-read alignment with read mismatch tolerance directed by Phred scores. The SAM files thus generated were converted to BAM format and sorted using SAMtools 1.21.

Sequencing duplicates, that were brought into the dataset with PCR amplification, were demarcated and eliminated using Picard (Mark Duplicates). Filtered, aligned BAM files were indexed to hasten the later variant calling procedure. Alignment figures (e.g., mean depth of coverage, proportion of aligned reads, duplications) were tabulated in each sample in order to authenticate data quality.

### 2.5. Variant Calling and Filtration

To ensure reliable identification of variants, the following two independent variant callers were used for SNP calling.

1. Genome Analysis Toolkit (GATK) Pipeline:
  ○ Base Quality Score Recalibration (BQSR): adjusts for systematic errors in base quality scores.
  ○ HaplotypeCaller: simultaneously identifies SNPs and small INDELs.
  ○ Genotype Refinement: generates a preliminary VCF file.
2. SAMtools:
  ○ mpileup: builds read alignments across the exome.
  ○ bcftools call: discerns SNPs and small INDELs, creating a separate VCF file.

To minimize false positives, only those variants identified by both pipelines—GATK and SAMtools—were retained. This cross-validation strategy markedly enhances confidence in variant calls. Furthermore, hard filters based on coverage depth (≥10 reads), variant quality (e.g., QUAL score ≥ 30), and other metrics (e.g., MQ, FS) were applied. Variants failing these criteria were removed. The resulting high-confidence variant set contained both coding SNPs (missense, nonsense, synonymous) and small INDELs, forming the basis for downstream analyses.

### 2.6. Functional Annotation and Impact Prediction

To classify variants into functional classes, the VCF files annotated were run through the SNPEff program, which assigns variants into high, moderate, low, or modifier impact based on their genomic feature (exonic, intronic, UTR), type of mutation (missense, nonsense, frameshift, etc.), and predicted functional effect. This preliminary classification gave a general idea of possible protein-altering events. To further determine the functional effect of non-synonymous coding SNPs, PolyPhen-2 (Polymorphism Phenotyping v2) and SIFT (Sorting Intolerant From Tolerant) were utilized. PolyPhen-2 assesses amino acid changes by combining protein sequence, structural, and functional annotations, producing a probabilistic score that classifies variants as benign, possibly damaging, or probably damaging. Variants with a score ≥ 0.85 were assigned “probably damaging”, those with a score between 0.15 and 0.85 as “possibly damaging”, and those with a score of less than 0.15 as “benign”.

SIFT, however, is based on a prediction of variant effects by comparing alignments of homologous protein sequences in order to quantify evolutionary conservation. It predicts a score of “tolerated” for >0.05 and “deleterious” for ≤0.05, according to the premise that substitutions at conserved sites are more likely to impair protein function. Prioritization in further analyses was only given to variants predicted as “damaging” by PolyPhen-2 and “deleterious” by SIFT since their intersection enhances confidence in their functional effect. This method utilizes two standalone computational approaches, each based on different predictive features like sequence conservation, biochemical properties, and structural modeling, to improve the accuracy of variant classification.

### 2.7. Prioritization of Genes of Interest

Given this study’s primary objective of identifying SNPs related to T2DM and possible renal complications, variants identified as damaging or deleterious in genes previously implicated in T2DM, insulin secretion, or kidney function were flagged for detailed investigation. These genes included but were not limited to

- INSR, ABCC8, KCNJ11, MAFA, ACE, IKBKB, TNF, MAPK1, MAPK8, CACNA1C, CACNA1D, PRKCD, PRKCZ, IRS2, SOCS1, and PIK3R3.

For each of these genes, the specific amino acid change, its location within the protein, and its associated PolyPhen-2/SIFT scores were tabulated (Table 2). Potential genotype–phenotype correlations (e.g., presence of kidney-specific complications in carriers) were noted.

### 2.8. Pathway Analysis and Gene Ontology (GO) Enrichment

To understand how damaging variants converge on metabolic and inflammatory pathways

1. KEGG Mapper was used to map gene IDs carrying high- or moderate-impact variants onto known canonical pathways, notably the T2D pathway.
2. Significant Pathways: genes related to insulin signaling (*INSR, IRS2, PIK3R3*), β-cell K_ATP channels (*ABCC8, KCNJ11*), and inflammation (*TNF, IKBKB*) were frequently enriched (q-value < 0.0001).

Gene Ontology classification was performed to elucidate broader biological processes (BPs), molecular functions (MFs), and cellular components (CCs) potentially disrupted:

✓ BPs: glucose homeostasis, lipid metabolism, and cytokine-mediated inflammatory responses.
✓ MFs: ATP binding, kinase activity, receptor ligand binding.
✓ CCs: plasma membrane receptors, intracellular signaling complexes.

Genes with multiple damaging variants in the same pathway or GO category were flagged as high-priority candidates for functional follow-up.

### 2.9. Statistical Analyses

A preliminary association test (e.g., chi-square or Fisher’s exact) was conducted to compare the frequency of selected damaging SNPs between the T2DM group and healthy controls. While sample size constraints limited the statistical power, observed trends provided clues regarding which alleles might be enriched in the T2DM population. Where available, clinical measures (e.g., fasting blood glucose, HbA1c, serum creatinine) were correlated with variant status. Participants carrying multiple high-impact variants in key insulin or renal genes were evaluated to see if they exhibited more advanced or earlier onset nephropathy.

### 2.10. Identification of PolyPhen-2 and SIFT Scores

Because a major goal was to pinpoint variants with genuine functional consequences, a central methodological step involved applying PolyPhen-2 and SIFT to all missense SNPs. The rationale and details are as follows:

1. PolyPhen-2
  ○ Input: for each missense SNP, the reference and alternate amino acids and their positions in the canonical protein sequence were submitted along with additional protein structure/function metadata.
  ○ Algorithm: PolyPhen-2 integrates multiple features—sequence conservation, presence/absence of structural domains, known functional residues, and evolutionary relationships among homologous proteins.
  ○ Result Interpretation:
    ▪ Benign: low likelihood of damaging protein structure/function.
    ▪ Possibly Damaging: intermediate level of confidence requires further validation.
    ▪ Probably Damaging: high confidence that the variant impairs normal protein function.
2. SIFT
  ○ Input: the same missense variant data, but with particular emphasis on alignments against sequences from multiple species to gauge evolutionary conservation.
  ○ Algorithm: SIFT calculates a normalized probability for each possible amino acid substitution at a given position. The more conserved the position, the higher the likelihood that a substitution will be deleterious.
  ○ Result Interpretation:
    ▪ Tolerated (score > 0.05): the substitution is less likely to impact protein function.
    ▪ Deleterious (score ≤ 0.05): the substitution is likely to disrupt the protein’s normal activity.
3. Intersection of Predictions
  ○ Stringent Criteria: variants that both PolyPhen-2 labeled as “probably damaging” and SIFT labeled as “deleterious” were prioritized in subsequent analyses of T2DM predisposition and renal complications.
  ○ Biological Relevance: positions flagged by both algorithms often represent evolutionarily conserved and structurally or functionally crucial residues.

Through these complementary methods, this study avoided overemphasizing variants predicted to be damaging by a single approach (thereby limiting false positives). Instead, it spotlighted “consensus damaging variants”, which are more likely to underlie the pathophysiological mechanisms of T2DM and diabetic nephropathy.

## 3. Result

This study population is composed of 26 DN patients (18 males and 8 females). Out of 26, 16 were aged greater than 60 years and 10 were aged less than 60 years. Based on renal function, patients were divided into two groups: controls, who had T2DM for more than ten years and had no renal impairment (UAE < 30 mg/g and eGFR > 60 mL/min/1.73 m^2^) and DN patients, including those with mild DN (UAE 30–300 mg/g and/or eGFR 30–59 mL/min/1.73 m^2^) and severe DN (eGFR 1 < 29 mL/min/1.73 m^2^ and/or UAE > 300 mg/g). In accordance with the American Diabetes Association’s (ADA) criteria, people were diagnosed with T2DM [28]. The presence of DN was assessed using urinary albumin excretion (UAE) levels and the estimated glomerular filtration rate (eGFR), which was computed using the Chronic Kidney Disease Epidemiology Collaboration (CKD-EPI) equation, in accordance with the Kidney Disease Improving Global Outcomes (KDIGO) guidelines [29].

Serum and plasma samples were collected after a 12 h fast for laboratory analysis. The Jaffe reaction was used to measure the levels of creatinine. Glycated hemoglobin (HbA1c) levels were assessed using several techniques, and the results may be linked to the Diabetes Control and Complications Trial approach by conversion formulas or offline calibration. Low-density lipoprotein (LDL) cholesterol was computed using the Friedewald formula, whereas triglycerides, total plasma cholesterol, and high-density lipoprotein (HDL) cholesterol were measured enzymatically. Two readings of blood pressure (BP) were taken using an Omron digital sphygmomanometer following a 5 min rest period while seated. Systolic blood pressure (SBP) and diastolic blood pressure (DBP) were computed using the mean of the two parameters. Hypertension was defined as blood pressure readings more than 140/90 mmHg or antihypertensive treatment.

### 3.1. Overview of Variant Identification and Classification

The objective of this study was to uncover genetic variants, particularly SNPs, that have a tendency to cause T2DM or complicate its course toward diabetic nephropathy. In this analysis based on WES information in 26 T2DM subjects (including diabetic nephropathy subjects) and control subjects, 9404 genetic variants were found. Out of them, 8725 (92.8%) were SNPs and 679 (7.2%) were INDELs.

Considering the significance of coding-region mutations to disease phenotypes, focus was on SNPs that change or can harm protein function. Toward this end, we combined two complementary silico prediction programs: PolyPhen-2 (which predicts SNPs to be “benign”, “possibly damaging”, or “probably damaging”) and SIFT (which predicts SNPs to be “tolerated” or “deleterious”). Variants identified as “damaging” or “deleterious” by both measures were given top priority for follow-up, on the premise that agreement of multiple algorithms indicates a high likelihood of functional effects.

Over 1000 SNPs were classified as damaging by PolyPhen-2, and more than 1800 SNPs were labeled as deleterious by SIFT. From the intersection of these calls, a final subset of 536 SNPs emerged as both damaging (PolyPhen-2) and deleterious (SIFT). These SNPs form the core set of candidate variants with the strongest likelihood of compromising normal protein structure and function.

### 3.2. Chromosomal Distribution of Variants

**Figure 1:**
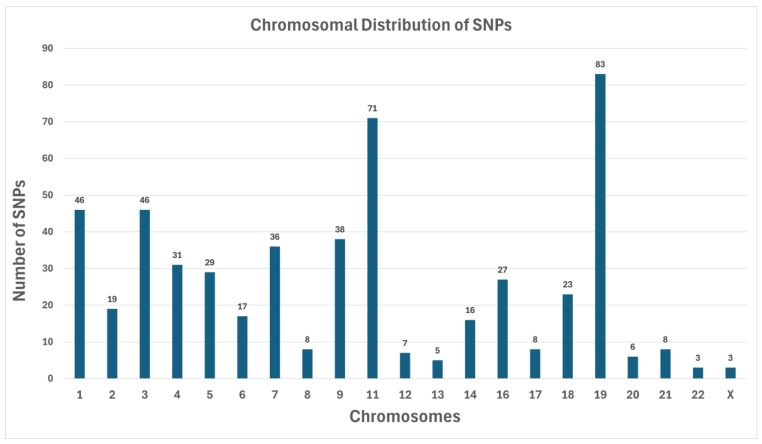
Chromosomal distribution of SNPs in T2DM patients. Distribution of identified SNPs across different chromosomes in the T2DM and nephropathy study populations. Chromosome 19 harbored the highest SNP count, while chromosomes 20, 21, 22, and X were found to have fewer variants. This distribution presumably represents the genomic regions associated with susceptibility to T2DM and progression to diabetic nephropathy.

To reveal patterns in how variants might be organized across the genome, the identified SNPs were mapped to their respective chromosomes (Figure 1). The distribution demonstrated both localized “hotspots” and comparatively sparse regions, reflecting the complexity of the genetic architecture underlying T2DM and its complications.

#### SNP Counts by Chromosome

- Chromosome 19 has the highest number of SNPs (83) (Figure 1). This observation is consistent with longstanding findings that Chromosome 19 is relatively gene-dense and carries many loci implicated in metabolic regulation.
- Chromosome 1 had 46 SNPs, along with Chromosome 3, which also had 46. Historically, Chromosome 1 is known to contain multiple diabetes-associated loci (e.g., regions near INSR).
- Chromosome 11 contained 71 SNPs, notable because it houses genes like KCNJ11 and ABCC8, both of which are critical for insulin secretion.
- A small number of chromosomes (e.g., Chromosomes 20, 21, 22, and X) showed minimal SNP counts, each having fewer than 10 identified variants in our filtered list. The lowest counts were Chromosome 22 (3 SNPs) and Chromosome X (3 SNPs), possibly reflecting either a lack of highly damaging variants in the coding regions for these chromosomes in our cohort or limited representation of X-linked metabolic disruptions in this sample set.

### 3.3. PolyPhen-2 and SIFT Score Distributions

**Figure 2:**
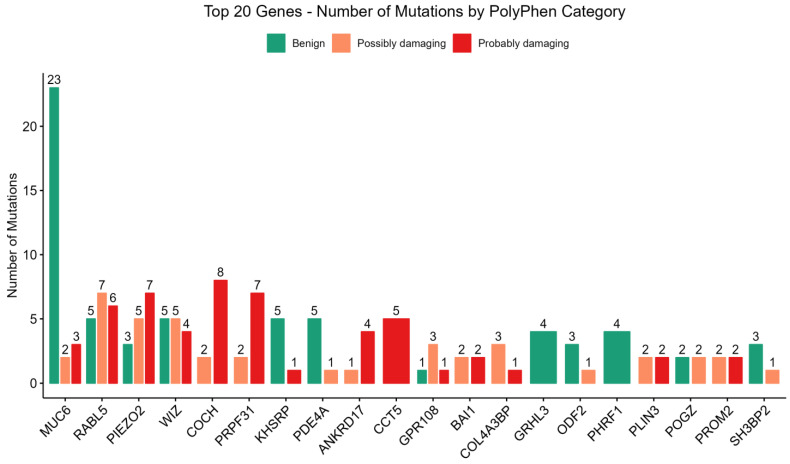
PolyPhen-2 predictions for SNPs identified in T2DM patients. The classified SNPs were predicted to have one of three impacts on their protein function: benign, possibly damaging, or probably damaging.

PolyPhen-2 predictions (Figure 2) were categorized into three general groups, viz., benign (B), possibly damaging (PD), and probably damaging (PrD). Analysis of predicted functional consequences of genetic mutations across a cohort revealed a spectrum of potential impacts, highlighting genes with a notable burden of variants predicted to alter protein function. Among the genes exhibiting the highest number of mutations, MUC6 showed the greatest frequency (n = 28). However, a significant majority of these MUC6 variants were functionally predicted as benign (n = 23) by PolyPhen-2, suggesting a limited impact on protein function despite the higher mutation count.

In contrast, several other genes displayed a substantial proportion of mutations predicted to be function-changing, categorized as possibly damaging (PD) or probably damaging (PrD). These genes, and their respective counts of predicted damaging mutations, include RABL5 (PD = 7, PrD = 6), PIEZO2 (PD = 5, PrD = 7), WIZ (PD = 5, PrD = 4), COCH (PD = 2, PrD = 8), and PRPF3 (PD = 3, PrD = 7). The enrichment of potentially deleterious variants within these genes underscores their potential roles in the pathogenesis of DN.

**Figure 3:**
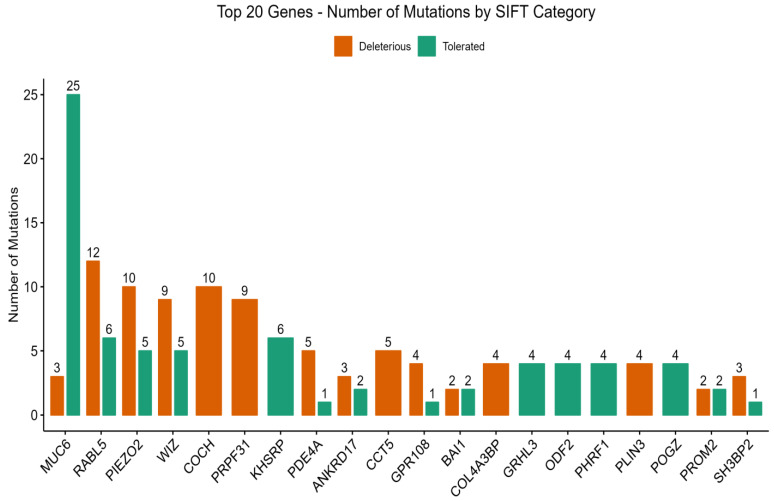
SIFT score distribution for SNPs in T2DM patients. Variants were classified as “tolerated” or “deleterious” based on their functional impact.

Functional consequences of observed genetic variants were further assessed utilizing the SIFT algorithm, which categorizes predictions as “tolerated” (T), indicating a low likelihood of impact on protein function, or “deleterious” (D), suggesting a considerable impairment of protein function. Consistent with the PolyPhen-2 analysis, the genes exhibiting the highest number of total mutations according to SIFT were MUC6 (n = 28), RABL5 (n = 18), PIEZO2 (n = 15), WIZ (n = 14), and COCH (n = 10) (Figure 3).

Focusing on variants predicted to impair protein function, the genes with the highest counts of “deleterious” mutations by SIFT included RABL5 (n = 12), PIEZO2 (n = 10), COCH (n = 10), WIZ (n = 9), PRPF31 (n = 9), and PDE4A (n = 5), among others. This highlights a shared set of genes predicted to harbor functionally significant variants by both algorithms, while also introducing additional candidates with a notable burden of predicted deleterious mutations.

Expanding on the potential implications of disrupted function for those genes prominently featuring in the SIFT deleterious predictions, like PRPF31 and PDE4A, reveals further connections to the complex pathology of DN.

### 3.4. Key Genes Linked to Type 2 Diabetes Mellitus (T2DM) and Diabetic Nephropathy (DN)

**Figure 4:**
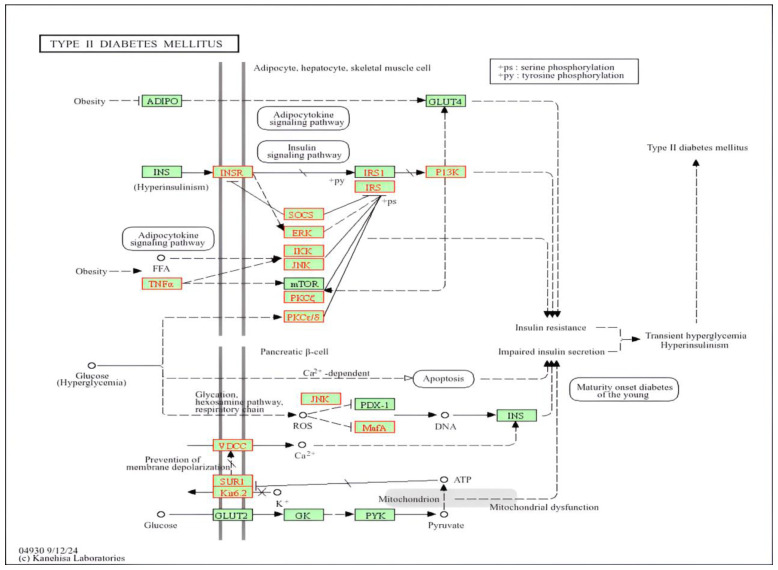
Key genes associated with T2DM pathophysiology and insulin resistance. Pictorially representing genes in this study implicated in T2DM. Genes highlighted in red with a red box outline indicate those with identified mutations, while genes in red with a black box outline represent genes affected by the mutated genes.

Analysis of gene functionality and referencing existing knowledge from the literature and databases such as KEGG (Kyoto Encyclopedia of Genes and Genomes), highlighted 15 particularly noteworthy genes (Table 2). Each of these genes plays well-defined roles in T2DM susceptibility or progression (Figure 4) and many have strong links to DN.

**Table 2:** List of high-impact variants identified in key T2DM-associated genes.

Gene	Location	Allele	Variant Type
*ABCC8*	11:17493912-17493912	TGTT	intron_variant
	12:2550682-2550682	C	intron_variant
*CACNA1D*	3:53848646-53848646	T	downstream_gene_variant
*IKBKB*	8:42156045-42156045	ATG	intron_variant
*INSR*	19:7293887-7293887	C	upstream_gene_variant
*IRS2*	13:110424850-110424850	A	intron_variant
*KCNJ11*	11:17415389-17415389	G	upstream_gene_variant
*MAFA*	8:144512253-144512253	G	synonymous_variant
	8:144517130-144517130	G	upstream_gene_variant
*MAPK1*	22:22162633-22162633	C	intron_variant
*MAPK8*	10:49515638-49515638	A	intron_variant
	10:49515970-49515970	T	intron_variant
	10:49610716-49610716	T	intron_variant
	10:49612299-49612299	A	intron_variant
	10:49614180-49614180	G	intron_variant
	10:49620460-49620460	A	downstream_gene_variant
	10:49632740-49632740	C	upstream_gene_variant
	10:49648606-49648606	A	downstream_gene_variant
*PIK3R3*	1:46547692-46547692	C	intron_variant
*PRKCD*	3:53189911-53189911	G	upstream_gene_variant
*PRKCZ*	1:2074301-2074301	C	upstream_gene_variant
*SOCS1*	16:11350612-11350612	T	upstream_gene_variant
*TNF*	6:31538847-31538847	C	upstream_gene_variant

### 3.5. Biological Pathway Involvement

Besides focusing on individual genes, we mapped high-impact and moderate-impact variants to the KEGG database. This approach allowed us to identify pathways that are likely dysregulated, with common threads linking T2DM and diabetic nephropathy:

a. Insulin Signaling: Insulin signaling includes genes INSR, IRS2, PIK3R3, PRKCZ, and various MAPK family members. Disruption in this axis will result in decreased glucose uptake, hyperglycemia, and increased lipolysis.
b. β-Cell Function and Insulin Secretion: The important gene is CACNA1C. Changes in IKBKB, SOCS1, and MAPK8 aggravate pro-inflammatory networks, thereby fueling systemic insulin resistance and provoking local renal inflammation. Such persistent low-grade inflammation disturbs homeostasis in the kidney and may speed the progression toward end-stage renal disease (ESRD).
c. Inflammatory Pathways: IKBKB, and some MAPK family members, highlight how immune regulators are intimately involved in the development of insulin resistance and the vascular inflammation that are the hallmark of diabetic complications. SOCS1 and MAPK8 aggravate pro-inflammatory networks.

### 3.6. Biological Processes Potentially Affected

The analysis of higher-level GO terms related to the mutated genes allowed us to better understand the multiorgan nature of T2DM and renal complications:

Glucose and Lipid Homeostasis: many of the genes identified (INSR, MAFA, for example) are involved in pathways maintaining euglycemia along with a balanced lipid homeostasis.

Oxidative Stress and Mitochondrial Function: T2DM is commonly associated with an elevated production of reactive oxygen species (ROS), due in part to hyperglycemia-induced flux through polyol and hexosamine pathways. Genes such as PRKCD can extend oxidative damage in cells, particularly regarding the kidney, whereas its vasculature is especially sensitive to variations in oxidative status.

### 3.7. Linking Genetic Findings to T2D Severity and Progression

One of the uncertainties in T2DM lies in the fact that, within the context of the heterogeneous clinical presentation among patients, some maintain glycemic control for many years while developing few complications, whereas others progress rapidly to nephropathy or some other complication-leading growth. Our results demonstrate how functionally damaging variant accumulation across multiple genes and pathways renders individuals more prone to a more aggressive course:

1. Polygenic Risk Load: a patient with damaging variants in both INSR and KCNJ11, for example, might experience concurring β-cell failure and insulin resistance, which will exacerbate hyperglycemia.
2. Inflammatory Burden: other variants in TNF or IKBKB could fuel an inflammatory cascade that may, in turn, damage micro-vessels.
3. Renal-Specific Dysregulation: opportunistically damaging variants at PRKCD, or certain MAPK genes, would additionally confer a renal stress element in an environment that dwells upon sugars, hence progressing kidney injury.

## 4. Discussion

The present study investigated the association of certain loci with diabetic nephropathy (DN) since identification of these loci will help in prevention, early diagnosis, and treatment of DN. The results indicated that there were variations in the *COCH* gene (Figure 2 and Figure 3). The *COCH* gene encodes the cochlin protein. This gene was reported among the risk genes of DN in T1DM [30].

Variations in *pre-mRNA processing factor 31 (PRPF31)* gene were also encountered (Figure 2 and Figure 3). It has been shown that *PRPF31* (+/−) knockout mice have elevated total body fat and increased fasting glucose and that a variant of *PRPF31* is associated with metformin response [31].

Results also indicated that there are variations in the *PIEZO2 gene* (Figure 2 and Figure 3), that encode PIEZO2 protein. The *PIEZO2* gene was reported to mediate the formation of glomerular fibronectin, which results in glomerulosclerosis and development of DN in the mice model [32]. In addition, the *PIEZO2* gene may be implicated in diabetic peripheral neuropathy and microangiopathy [33]. In addition, data indicated that there were variations in the *RABL5* gene (Figure 2 and Figure 3). This gene is also called IFT22 intraflagellar transport 22 (*IFT22*). The *IFT22* gene supports ciliary function, and it is one of the ciliary Rab-like small GTPases which play important regulatory roles in ciliary BBSome transport [34]. The BBSome is a complex of octameric protein that regulates the transport and signaling of cilia [35]. The *IFT22* gene is implicated in autosomal recessive polycystic kidney disease (ARPKD) [36] and Bardet-Biedl syndrome (BBS) [37].

There were also variations in the *CCT5* gene (Figure 2 and Figure 3); this gene encodes the chaperonin containing TCP1 subunit 5. The CCT5 gene variations were reported to be associated with neuropathy [38,39]. In addition, data indicated that there were variations in the ANKRD17 gene (Figure 2 and Figure 3). The *ANKRD17* gene encodes an ankyrin repeat-containing protein, this protein was reported to be involved in cell cycle progression. The ANKRD17 gene variation was reported to cause a neurodevelopmental disease [40].

The *WIZ* (Widely Interspaced Zinc Finger) gene also exhibited variations (Figure 2 and Figure 3). It encodes the WIZ, a zinc finger protein which is important for craniofacial development [41] and is upregulated in hepatocellular carcinoma [42].

Results indicated that there are variations in the MUC6 gene (Figure 2 and Figure 3). The MUC6 gene is expressed in the tissue of the stomach and pancreas, is linked to hypertrophic cardiomyopathy, and enhances innate immune reactivity and DM [43,44]. The MUC6 gene was reported among the candidate genes implicated in T1DM [44]. In addition, missense mutation in MUC6 gene contributes to the development of pulmonary artery hypertension [45]. There were also variations in BAI1 (Figure 2 and Figure 3). This gene is also known as adhesion G protein-coupled receptor B1 (ADGRB1). The ADGRB1 (BAI1) gene was reported to be associated with susceptibility to seizure and altered brain development [46].

The *PLIN3* gene also showed variations (Figure 2 and Figure 3). It has been shown that the circulating *PLIN3* is linked with insulin resistance in T2DM patients [47]. The *Prominin 2 (PROM2)* gene also exhibited variations (Figure 2 and Figure 3). This gene is associated with ovarian cancer, lung cancer, and renal disease [48,49].

Results indicated that there is a variation in the *KHSRP* (KH-Type Splicing Regulatory Protein) gene (Figure 2 and Figure 3). The *KHSRP* gene encodes a protein that binds the single-stranded nucleic acid [50]. It regulates gene expression and RNA life on multiple levels [50]. KHSRP regulates a variety of critical cellular processes, including metabolism, differentiation, proliferation, and infection response [50]. It has been reported that there is a potential association between the changes in *KHSRP* function or expression and disorders such as obesity, T2DM and cancer [50]. It has been reported that the increased expression of the *KHSRP* gene prevents pancreatic beta cell death and enhances the secretion of insulin, whereas reduced KHSRP gene expression results in increased pancreatic beta cell death and defective insulin secretion [51]. Moreover, the *KHSRP* gene was reported to be involved in type 2 diabetic skin [52].

The *COL4A3BP* gene also exhibited a variation (Figure 2 and Figure 3). This gene was reported to be associated with metabolic diseases [53]. There was also mutation in *GPR108* (Figure 2 and Figure 3); this gene encodes the G protein-coupled receptor 108. The GPR108 was described as a potent NF-κB activator, and it was reported to be involved in a signal transduction activated by the Toll-like receptors (TLRs) [54]. The NF-κB pathway was reported to have a role in the development of DN [55]. The TLRs were described to be implicated in DM and diabetes complications [56,57].

Results indicated that there are variations in the *PDE4A* gene (Figure 2 and Figure 3) that encodes for the 3′,5′-cyclic-AMP phosphodiesterase 4A. The PDE4 family is considered important for the regulation of cAMP in pancreatic beta cells [58], and the inhibitor of PDE4 alleviates the symptoms of DM by improving the increased blood sugar and insulin resistance [59]. Moreover, PDE4 inhibitors are beneficial for DN treatment since the PDE4 inhibitors suppress oxidative stress, nephritis, and renal fibrosis [59]. In addition, the PDE4 family is implicated in cardiac dysfunction associated with DM [60], and the expression of *PDE4A* is significantly decreased in the explanted hearts from cases with idiopathic dilated cardiomyopathy in comparison with healthy controls [60]. The role of PDE4A in diabetic complications remains to be investigated in future studies.

The data also showed variations in the *SH3BP2* gene (Figure 2 and Figure 3), this gene encodes the SH3BP2 protein which is implicated in nephrotic syndrome [61].

The key genes linked to T2DM and DN (Table 2) include the *CACNA1D (Calcium Voltage-Gated* Channel Subunit Alpha1 D) gene. This gene encodes for the primary L-type voltage-gated calcium channel (Ca_v_1.3) in the insulin-producing pancreatic beta cells [62], and CACNA1D variants probably contribute to the development of T2DM [63].

We also detected variations in the *IKBKB* (Inhibitor of Nuclear Factor Kappa-B Kinase Subunit Beta) gene (Table 2). The IKBKB was identified as a gene implicated in the crosstalk during mitochondrial and insulin signaling [64]. Moreover, the NF-κB pathway is reported as a potential target for the treatment or prevention of DM and DN [55,65].

Variations in the *IRS2* (insulin receptor substrate 2) gene were also found (Table 2), (Figure 4). The *IRS2* gene encodes insulin receptor substrate 2. It has been suggested that in obesity and T2DM the IRS2-mediated insulin signaling is impaired in hepatocytes [66]. Moreover, in golden (Syrian) hamster, it has been demonstrated that the defective IRS2 results in non-obese T2DM with pancreatic beta cell β-cell dysfunction [67].

The *MAFA* (MAF bZIP transcription factor A) gene showed variations (Table 2). MAFA is a pancreatic beta-cell-enriched transcription factor that is necessary for beta cell differentiation and maturity [68]. It also controls the expression of other genes linked to beta cell function, such as those that sense glucose and produce and exocytose insulin [68]. It was reported that there is no expression of the MAFA gene in islets of Langerhans from patients with T2DM contributing to pancreatic-cell dysfunction observed in T2DM [69]. Furthermore, studies have shown that mutations in the *MAFA* gene result in DM [70]. Furthermore, *MAFA* may be involved in lipid metabolism and differentiation of fat cells via the adipocytokine network in pancreas [71].

Variations in the mitogen-activated protein kinase 1 and 8 (*MAPK1* and 8) gene were also detected (Table 2). The MAPK signal transduction pathway is probably implicated in the destruction of pancreatic beta cells through the inflammatory cytokine, interleukin-1 beta [72]. The MAPK pathway may also be implicated in insulin resistance [73]. Moreover, it has been reported that MAPK1 mediates the disruption of mitochondria-associated endoplasmic reticulum membrane (MAM), as well as the dysfunction of mitochondria in DN through the phosphofurin acidic cluster sorting protein 2 (PACS-2)-dependent mechanism [74]. Furthermore, *MAPK8*, regulates various physiological processes such as inflammation, cell differentiation, proliferation, and death. The defective MAPK8 has also been involved in several diseases, for instance, DM and cancer [75]. In addition, it has been reported that there is a dysregulation of the MAPK signaling pathway in acute and chronic renal disease [76].

The data indicated that there are variations in the phosphoinositide-3-Kinase Regulatory Subunit 3 (PIK3R3) gene (Table 2) (Figure 4). The PIK3R3-HNF4α-PPARα signaling axis has an important role in lipid metabolism in hepatocytes, and the activation of PIK3R3 reduces hepatosteatosis; thus, PIK3R3 can be regarded as a promising novel target for NAFLD and metabolic syndrome treatment [77]. The metabolic syndrome is linked with T2DM [78].

There were also variations in PRKCD (protein kinase C delta or PKC-δ) and PRKCZ (protein kinase C zeta type or PKCζ) genes (Table 2) (Figure 4). Protein kinase C regulates multiple processes in the pancreas, and its dysregulation is linked to pancreatic pathologies [79]. Moreover, the activation of PKC isoforms has been implicated in DN pathogenesis [80]. The expression of the PRKCD gene is increased in obese people and was positively correlated with fasting glucose and blood triacyclglycerols [81]. In addition, PRKCD is implicated in intracellular accumulation of ROS, leading to oxidative stress and insulin resistance in adipose tissues [82], while there is defective PRKCZ activity and glucose uptake (insulin resistance) in muscle and adipose tissues in T2DM cases [83,84]. Moreover, it has been reported that PKC is involved in the development of microvascular complications of DM including DN [85].

Variations in suppressors of the cytokine signaling-1 (*SOCS-1*) gene were also found (Table 2). SOCS-1 is involved in inflammatory response and insulin resistance [86]. It has been shown that there is an increased *SOCS-1* expression in insulin-sensitive tissues in obesity [87,88] and that this inhibits the phosphorylation of IRS-1 and IRS-2, leading to the inhibition of downstream signaling and insulin resistance [87,88].

Limitations of the present study include the relatively small sample size, and the effect of these variations requires further investigations. Future studies with larger sample size and protein functional studies [89,90,91] are warranted to validate these findings. Thereafter, these loci can be utilized in genetic testing for the identification of individuals or populations at risk of DM and DN.

## 5. Conclusions

In summary, in the present study, the association of genetic loci with diabetic nephropathy (NP) was investigated in Saudi patients using whole exome sequencing (WES) and bioinformatics analysis. Results indicated that there were variations in certain genes including *COCH*, *PRPF31*, *PIEZO2*, *RABL5*, *CCT5*, *PLIN3*, *PDE4A*, *SH3BP2*, *MUC6*, *CACNA1D*, and *MAFA*. The processes that are potentially influenced by these gene variations include insulin signaling and secretion, the inflammatory pathway, and mitochondrial function. These results help in the identification of the genes involved in diabetes mellitus and DN, and after being verified in further studies, they can be utilized in detection and identification of susceptible individuals or populations for prevention and therapeutic strategies.

## Figures and Tables

**Table 1:** The clinical characteristics of DN cases included in this study.

Characteristic	N = 26 ^1^
Age (Years)	63 (59, 66)
Age Groups	
>60 Years	16 (61.53%)
40–60 Years	10 (38.46%)
Gender	
Female	8 (30.76%)
Male	18 (69.23%)
Duration of T2D (Years)	9.00 (7.00, 10.00)
Blood Sugar (Fasting)	156 (145, 160)
HBA1c (%)	7.40 (7.05, 7.50)
HBA1c Groups	
Diabetic	24 (90.30%)
Control diabetic	2 (7.69%)
BMI (kg/m^2^)	27.68 (22.24, 30.05)
BMI Groups	
Normal	10 (38.46%)
Obese	6 (23.07%)
Overweight	10 (38.46%)
Triglyceride (mg/dL)	210 (189, 242)
Triglyceride Levels	
<150 mg/dL	2 (7.70%)
≥150 mg/dL	24 (92.30%)
Total cholesterol (mg/dL)	
<200 mg/dL	12(46.15%)
>200 mg/dL	14(53.84%)
HDL (mg/dL)	34 (29, 48)
<40 mg/dL	15 (57.69%)
≥40 mg/dL	11 (42.30%)
LDL (mg/dL)	
>190 mg/dl	13 (50%)
160–190 mg/dL	10 (38.46%)
100–160 mg/dL	03 (11.53%)
VLDL (mg/dL)	
>40 mg/dL	10 (38.46%)
5–40 mg/dL	16 (61.53%)
Creatinine (mg/dL)	2.50 (1.90, 2.60)
BILIRUBIN (mg/dL)	1 (0, 2)
AST (U/L)	30 (20, 40)
ALT (U/L)	42 (37, 48)
ALP (U/L)	97 (73, 110)
Estimated glomerular filtration rate;	
eGFR, mL/min per 1.73 m^2^	41.0 (14.0–59.0)
Urinary albumin excretion (UAE)	
UAE, mg/g	90.5 (35.8–388.7)
30 mg/g	0
>30 to 300 mg/g	26
Arterial hypertension, %	90.3
Blood pressure	
SBP [mmHg]	150.0 [140.3–162.0]
DBP [mmHg]	87.0 [78.0–92.0]

^1^ Median (IQR); N (%); eGFR is estimated glomerular filtration rate; UAE is urinary albumin excretion. SBP is systolic blood pressure; DBP is diastolic blood pressure.

## Data Availability

All data connected with this research study are presented in the manuscript.
